# Cavernous hemangioma of mesentery involving the jejunum causing intestinal obstruction in an adult male

**DOI:** 10.1002/ccr3.5905

**Published:** 2022-05-27

**Authors:** Srijana Thapa, Udaya Koirala, Bijendra Dhoj Joshi, Anish Baniya, Bidushi Pokhrel

**Affiliations:** ^1^ Department of General Surgery Kathmandu Model Hospital Kathmandu Nepal; ^2^ Hospital for Advanced Medicine and Surgery Kathmandu Nepal

**Keywords:** cavernous hemangioma, intestinal obstruction, jejunum, mesentery

## Abstract

We present a rare case of a 45‐year‐old man with abdominal pain and features suggestive of intestinal obstruction. The CT scan of the abdomen demonstrated a large mesenteric mass involving the distal segment of jejunum. Surgical excision of the lesion and histopathological examination revealed the diagnosis of cavernous hemangioma.

## Graphical abstract

Gastrointestinal hemangiomas are uncommon and the presentation of the patient depends on the location of the tumor. Diagnosis is usually established following surgery and histopathology. Surgical resection is usually curative.

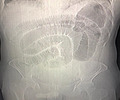

## 1 INTRODUCTION

Hemangiomas are benign tumors and can be present in any part of the body. The most common sites are skin, face, scalp, and back with frequent presentations involving other locations like liver, spleen, brain, spinal cord, adrenal glands, and mediastinum. However, gastrointestinal (GI) hemangiomas are uncommon.^1^, ^2^ There is no gender predominance, and the age of presentation varies from 2 months to 79 years.^3^ GI symptoms can vary according to the location, the most common being hematochezia.

A case of cavernous hemangioma being an unusual but potential cause of intestinal obstruction is hence presented, where the diagnosis was uncertain even after resection of the mass. Due to its rarity, diagnosis is often delayed. The definitive treatment entails resecting the mass. This case has been reported in line with the SCARE criteria.^4^

## 2 CASE REPORT

A 45‐year‐old man presented in the emergency department with generalized abdominal pain for 4 days and multiple episodes of vomiting for 1 day. He reported not having any bowel movement for 3 days, which was followed by four episodes of watery stool on the day of presentation. There was no history of melena or hematochezia. The patient had a smoking history of 3 pack‐years. His previous medical, surgical, and medication history were unremarkable.

On examination, the patient's blood pressure was 110/80 mm Hg with the heart rate of 88 beats per minute. The abdomen was uniformly distended and tenderness was elicited on deep palpation with no obvious mass. There was resonance on percussion and the bowel sounds were hyperactive. The hernial orifices were intact and the scrotal examination was unremarkable. The digital rectal examination showed an empty rectum. The rest of the systemic examination was unremarkable.

**FIGURE 1:**
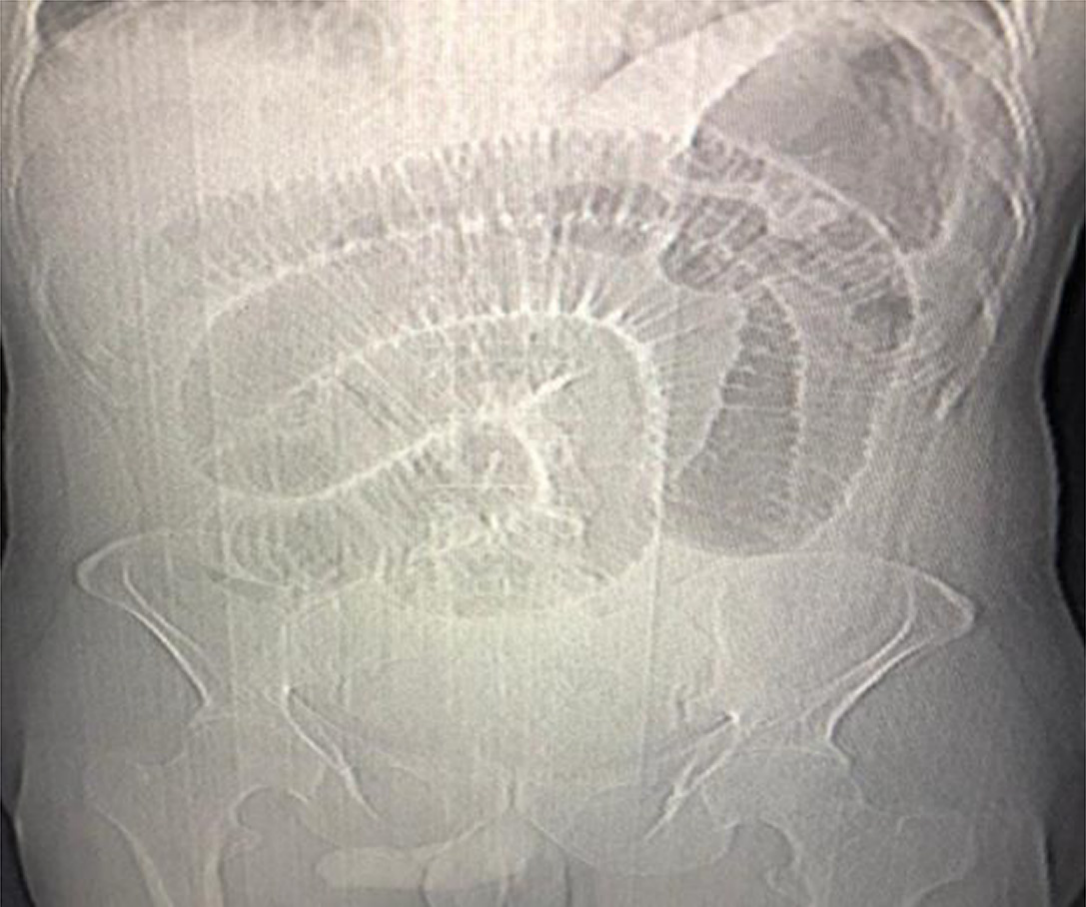
Abdominal X‐ray showing distended jejunal loops

**FIGURE 2:**
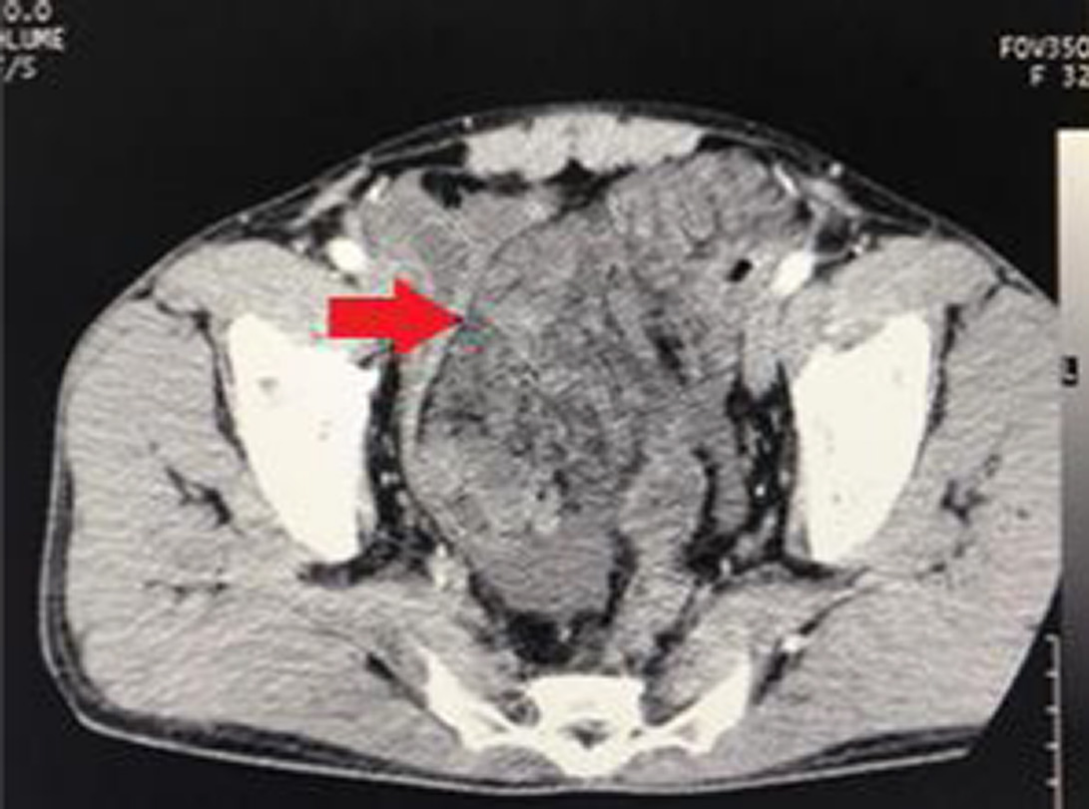
CT scan showing a huge mesenteric mass (arrow) in the pelvic region

On initial presentation, the patient's hemoglobin was 14.8 g/dl with white cell counts of 15,200/mm^3^ and C‐reactive protein (CRP) of 27.2 mg/L. The radiographic examination of the abdomen demonstrated distended jejunal loops (Figure 1). Ultrasonography was performed which showed distended bowel loops but revealed no other specific findings. The patient first opted for conservative management with intravenous fluids, antibiotic, gastric decompression, and urinary catheterization. Even after the initial management, his symptoms aggravated with increasing abdominal distension and discomfort; therefore, a computed tomography (CT) scan of the abdomen was performed. It demonstrated a large mesenteric mass measuring 12 cm × 6.7 cm in the pelvic region, encasing the long segment of distal jejunum with minimal circumferential enhancement and thickening of the jejunal wall (Figure 2).

**FIGURE 3:**
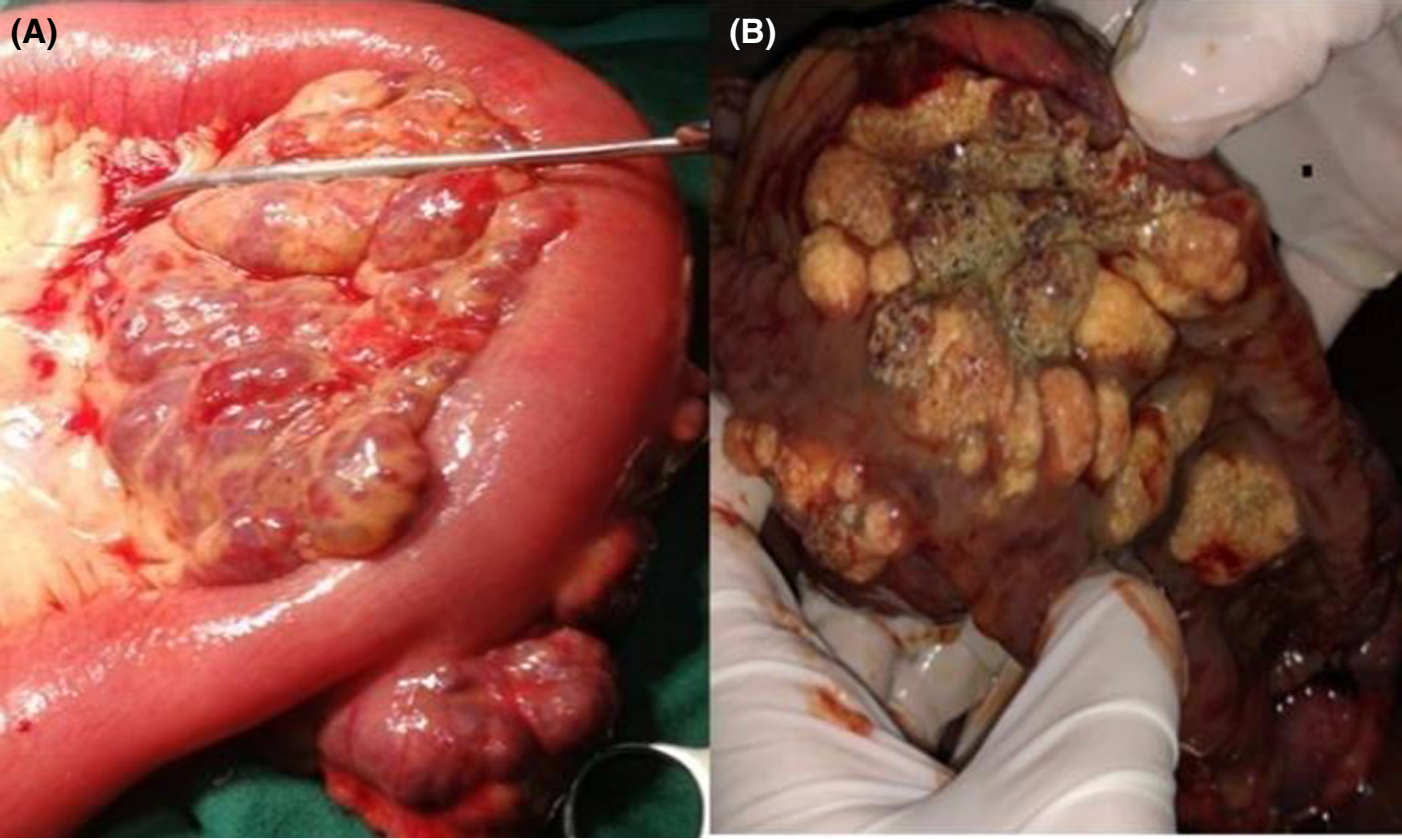
(A) Gross findings of the mesenteric mass on laparotomy (B) Cut section after incising the lumen of jejunum demonstrating multiple, yellowish nodular lesions circumferentially involving the jejunum and causing complete obstruction of the lumen

**FIGURE 4:**
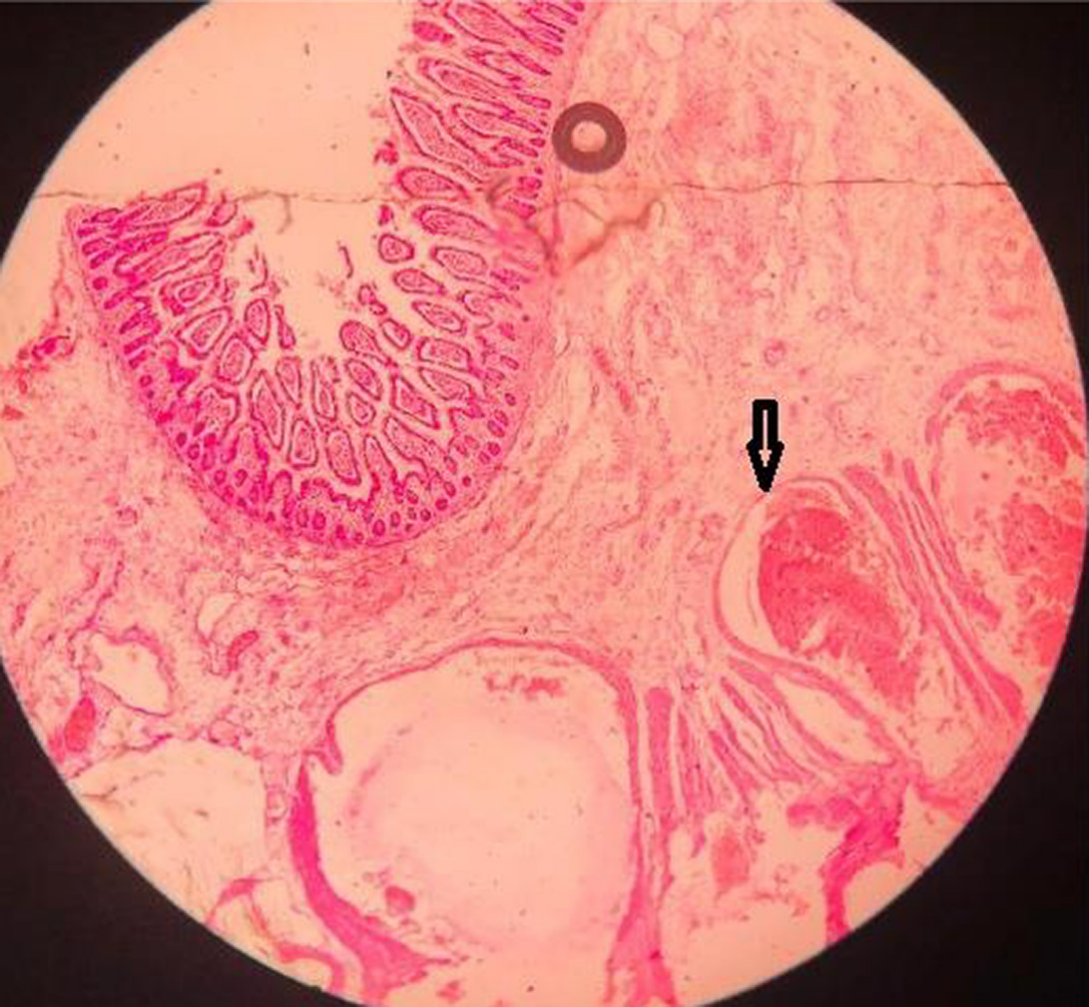
Numerous thin‐walled vascular channels lined by endothelium with red blood cells inside the lumen of the dilated channels (arrow)

An exploratory laparotomy was performed which revealed a large multi‐nodular mesenteric mass, bluish‐purple in color, and extending into the lumen of the jejunum. The involved jejunum was about 180 cms from the duodeno‐jejunal flexure and had multiple polypoidal yellowish lesions which apparently had caused complete obstruction of the lumen (Figure 3). The mesenteric mass along with the affected part of jejunum was resected and a side‐to‐side jejuno‐jejunostomy was performed. A 24 Fr abdominal drain was placed. The post‐operative evolution was uneventful. The subsequent histopathological report showed features compatible with cavernous hemangioma (Figure 4). The patient's follow‐up visits after 1 month and 1 year were unremarkable.

## 3 DISCUSSION

Mesenteric hemangiomas are very rare tumors of the gastrointestinal tract,^1^ and those involving the jejunum are even rarer. In the literature, isolated jejunal hemangiomas have been reported^5^, ^6^; however, large hemangiomas of mesentery involving the jejunum have not been reported till date. Cases of gastrointestinal hemangiomas involving the rectum,^7^ recto‐sigmoid junction,^8^ small bowel and mesentery,^3^ lesser omentum,^9^ and gastro‐splenic ligament^2^ have been identified. The most frequent occurrence of gastrointestinal hemangioma is in the small bowel followed by the colon.^8^ We herein have reported the case of an adult patient with a giant mesenteric cavernous hemangioma whose primary presentation was that of bowel obstruction; however, in general, the presentation can vary according to the site of the mass. Commonly, patients present with hematochezia, but melena, anemia, abdominal pain, dyspepsia, perforation, or intussusception have also been observed frequently.^8^

Abrahamson and Shandling have divided gastrointestinal hemangiomas into three types—namely capillary, cavernous, and mixed.^10^ Microscopic examination of cavernous hemangioma shows large dilated thin‐walled vessels with blood‐filled spaces. Cavernous hemangiomas are the most common type affecting the mesentery.^10^ Yang et al. have reported a hemangioma of cavernous and venous mixed type involving only the mesentery.^1^ In cases where both the mesentery and bowel are affected, identifying the origin of the hemangioma is difficult.^3^ Similarly, in our case, it was difficult to establish the origin of the hemangioma.

The diagnosis of mesenteric hemangiomas can be challenging and sometimes definitive diagnosis is only established upon histopathology. Due to the high risk of life‐threatening hemorrhage, biopsy of clinically suspected hemangiomas is strongly discouraged. However, CT scan and Magnetic Resonance Imaging (MRI) are useful modalities for diagnosis. CT scan usually demonstrates non‐homogeneous enhancement of lesion with transmural thickening of walls of the intestine involved.^11^ In our case, CT scan was suggestive of a huge mesenteric mass with distal jejunal wall thickening. MRI findings frequently demonstrate high signal intensity lesion on T_2_‐weighted sequences and low intensity on diffusion‐weighted MRI.^12^ MRI was not performed in our patient and we opted for surgical exploration.

The treatment of GI hemangiomas is surgical excision and likewise, in our patient, exploratory laparotomy with excision of the mesentery and affected bowel loop was performed. However, if a diagnosis had been made preoperatively, laparoscopic excision of the affected part could also have been performed.^13^

Mesenteric hemangiomas involving the bowel, despite being extremely rare, should be taken into account as potential diagnoses in patients presenting with features of intestinal obstruction. Sometimes, waiting for a definitive diagnosis can be lethal for patients' outcome, thus mandating an early intervention.

## AUTHOR CONTRIBUTION

ST, UK, and BDJ were entirely involved in the management of the patient and conception of the study. ST, AB, and BP prepared the manuscript. All authors were involved in critical revision of the manuscript.

## CONFLICT OF INTEREST

None.

## ETHICAL APPROVAL

None.

## CONSENT

Written informed consent was obtained from the patient to publish this report in accordance with the journal's patient consent policy.

## Data Availability

The data that support the findings of this study are available from the corresponding author upon reasonable request.
